# Recurring Transient Tooth Pain as Newly Described Symptom of Migratory Loiasis: A Mixed-Methods Study in Rural Gabon

**DOI:** 10.4269/ajtmh.24-0059

**Published:** 2024-07-23

**Authors:** Teite Rebecca Hildebrandt, Helmut Ramharter, Anita Lumeka Kabwende, Lilian Endamne, Saskia D. Davi, Ayôla Akim Adegnika, Ghyslain Mombo-Ngoma, Selidji T. Agnandji, Johannes Mischlinger, Rella Manego Zoleko, Michael Ramharter

**Affiliations:** ^1^ Centre de Recherches Médicales de Lambaréné, Lambarene, Gabon;; ^2^ Center for Tropical Medicine, Bernhard Nocht Institute for Tropical Medicine and I. Department of Medicine University Medical Center Hamburg-Eppendorf, Hamburg, Germany;; ^3^ Institute for Tropical Medicine, University of Tübingen, Tübingen, Germany;; ^4^ German Center for Infection Research, Partner Site Hamburg-Lübeck-Borstel-Riems, Germany

## Abstract

Loiasis, a filarial pathogen exclusively endemic in central and western Africa, causes a wide spectrum of symptoms. Understanding the breadth of its clinical manifestations is of importance for adequate patient care and to understand its disease burden. Recurring transient pain in the oral cavity was reported as a self-perceived symptom of loiasis in in-depth interviews of patients in a high transmission region in Gabon. Pain was described as stabbing in character and transient for a few days in its temporal course. A quantitative epidemiological survey indicated that transient tooth pain was experienced by 22% of patients infected with *Loa loa*. Among those individuals, it was exclusively reported by patients suffering from migratory loiasis (24%). Similar findings have been previously described for other filarial pathogens, indicating that transient swellings of the periodontium and the soft tissue of the oral cavity may explain this symptom reported by patients with migratory loiasis.

Oral diseases are among the most prevalent conditions both globally and regionally in sub-Saharan Africa, causing significant disease burden. Although two of the six main causes of oral disease are the specific dental diseases dental caries and periodontal disease, oral cancer, orodental trauma, and cleft lip and palate are general diseases of the oral cavity. Yet another two are caused by the infectious diseases HIV and noma.^1^ Several other infectious agents are known to manifest in the oral cavity with unspecific or pathognomonic signs. Viral, bacterial, fungal, and protozoan pathogens have been reported to cause or manifest in the oral cavity.^2^ However, to date little is known about helminthic pathogens and their impact on oral health.

*Loa loa* is a highly neglected filarial pathogen endemic in the rural forest and savannah regions of central and parts of western Africa, affecting more than 20 million people in medium to high transmission regions.^3^^,^^4^ Although loiasis has been neglected for decades as a rather benign infection, its considerable morbidity and mortality have been described recently, highlighting the important burden of disease on affected communities.^5^^–^^7^ Loiasis has a highly variable clinical penetrance, ranging from asymptomatic carriers, to the presence of acute or chronic signs and symptoms, to life-threatening complications such as encephalitis and renal and cardiovascular disease.^3^ The migration of the adult worm through the conjunctiva of the eye and localized subcutaneous transient swellings termed Calabar swelling are pathognomonic signs of loiasis, but many other symptoms are rather unspecific. These include chronic pruritus, myalgia, arthralgia, severe headaches, asthenia, and other atypical signs and symptoms.^3^^,^^8^ However, to date it remains unclear whether other, yet unreported disease manifestations consistently occur. In this article, we report recurring transient tooth pain as a newly described manifestation of loiasis.

In a qualitative study on the knowledge, attitudes, practices, and perceptions of loiasis in a highly endemic region of rural Gabon conducted between 2018 and 2020, in-depth interviews employing open-ended questions were performed with patients known to suffer from loiasis. Interviews were performed until saturation was observed for key outcomes. Participants were interviewed for all disease manifestations they experienced, and transient tooth pain was reported several times as one of the symptoms of loiasis. Tooth pain was reported to be located in the jaw and was characterized as stabbing without any other physical correlate. One participant described it by the analogy, “les vers creusent les dents,” indicating piercing pain of the jaw and teeth in the respective dental quadrant. Pain was almost always restricted to one quadrant and most of the time was even more localized. The pain gradually increased over a period of hours and resolved spontaneously without further medical intervention within a few days until it reappeared at a different location in the oral cavity. Several participants indicated a temporal proximity of the occurrence of transient tooth pain with eye-worm migration where one symptom commonly preceded the other.

In a second step, a quantitative survey of knowledge, attitudes, and practices was performed in the community Sindara, Ngounie province, in central Gabon in 2022 (institutional ethics committee of CERMEL approval no. CEI-008/2021).^9^ This village is characterized by one of the highest reported prevalences of loiasis in the endemic world.^6^ One hundred fifty adult villagers participated in semistructured interviews, which included specific questions about the presence of transient tooth pain. Trained field workers conducted the interviews with closed- and open-ended questions of a prevalidated questionnaire and coded the responses on a paper-based case record form. Prevalidation was performed by piloting of the questionnaire with independent interviewers before the main survey. In parallel, clinical and parasitological diagnostics for loiasis were performed using the WHO RapLoa questionnaire and a parasitological examination of midday peripheral blood for the detection of *L. loa* microfilariae.^3^^,^^10^^–^^13^ On the basis of these tests, participants were classified as migratory loiasis (defined as positive in RapLoa questionnaire irrespective of the presence or absence of peripheral microfilariae), non-migratory filariasis (defined as negative in RapLoa questionnaire but positive for microfilaremia), or uninfected persons (defined as negative in both RapLoa and microscopy).^14^

A total of 150 participants answered the question about the presence of recurring transient tooth pain. Twenty-seven individuals (18%) reported having experienced this symptom. The proportion of participants reporting the symptom was 10% (five of 51 participants) in the uninfected individuals and 22% (22 of 99 participants) in the *L. loa*–infected patients (odds ratio: 2.63; 95% CI: 0.92–7.54). Upon categorization into nonmigratory and migratory loiasis, the group proportions diverged further with 0% (zero of seven) of participants with nonmigratory loiasis and 24% (22/92) of patients with migratory loiasis reporting tooth pain as symptom, respectively.

This mixed-methods study provides the first evidence for recurring transient tooth pain as a newly described symptom of loiasis. Whereas the qualitative survey led to the identification of tooth pain as a potentially new clinical manifestation, the quantitative survey suggests that there might be an epidemiological causal link. It is striking that 24% of migratory loiasis patients reported this symptom compared with 0% of nonmigratory and 10% of uninfected participants, respectively, providing the first epidemiological evidence suggestive of a causal link between recurring transient tooth pain and migratory loiasis.

The finding of transient tooth pain being reported exclusively by patients with migratory loiasis corresponds well with findings from the qualitative survey. Migratory loiasis is defined as the report of a history of eye-worm migration and indicates signs and symptoms ascribed to the adult worm migration through the human body. It was previously shown that specific symptoms such as eye-worm migration and Calabar swelling as well as unspecific signs and symptoms such as generalized pruritus, arthralgia, myalgia, severe headaches, transient nerve palsies, transient vision loss, and asthenia are most closely linked to adult worm migration.^14^ Whereas the ocular symptoms of loiasis are directly caused by the migration of the worm through the eye, Calabar swelling is thought to be mediated indirectly by a hypersensitivity reaction to the migrating adult worm with subsequent angioedema.^15^ In contrast, the presence or extent of microfilaremia is not associated with a higher prevalence or intensity of specific or unspecific symptoms of loiasis.^3^^,^^14^

This finding corresponds well to the findings of this study indicating that transient tooth pain may be directly or indirectly caused in the orofacial region by the adult worm migration. Similar findings have been previously reported for other filarial pathogens.^16^^–^^18^ Given the transient nature of the reported symptom, the proximity to the anatomic predilection site of *L. loa* in the orofacial region, and its analogy with other filarial pathogens, we hypothesize that the transient tooth pain reported here shares the same pathophysiological mechanism.

This first report of recurrent transient tooth pain as a clinical symptom associated with loiasis is not without limitations. A main shortcoming is the lack of a thorough clinical and radiological dental examination in the cross-sectional survey, which was beyond the scope of this pilot study. Although a dental examination was not performed for all participants, the oral cavity of selected patients of the qualitative study was assessed by a physician. No evidence for other causes of dental disease was observed in this limited number of participants. It may also be speculated whether the reported tooth pain may more appropriately be classified as jaw pain or oral pain depending on the main site of inflammation. However, because patients consistently reported the symptom as tooth pain, we are confident using this patient-reported terminology. The study was also slightly underpowered to demonstrate statistically with a frequentist approach that tooth pain was associated with loiasis at α = 0.05. Despite the fact that an association was not demonstrated in this pilot study with statistical certainty, the disproportionately frequent reporting of transient tooth pain in loiasis patients and the exclusive reporting of this symptom in the migratory loiasis subpopulation supports a causal relationship. Future studies will have to include dedicated orodental investigations and imaging modalities to rule out other potential causes of tooth pain, including dental caries, dental and periodontal bacterial infections, orodental trauma, neuralgic pain, or local tumors as differential diagnosis of transient tooth pain.^2^ However, the consistency between the signs and symptoms reported in the qualitative survey and the clear epidemiological evidence in the quantitative study, paired with a comprehensible pathophysiology that is in line with the reported temporal characteristics and clinical symptoms, are a strength of this pilot study and provide initial evidence for a potential causal link between transient tooth pain and loiasis.

In conclusion, this mixed-methods study provides first evidence that transient tooth pain may be a frequent clinical manifestation of migratory loiasis in this highly endemic region. This preliminary finding requires further corroboration by scrupulously designed epidemiological studies of sufficient sample size in different geographic regions to confirm or refute this observation. Decisively answering this question will ultimately help provide better orodental care for patients in regions of high *L. loa* transmission by identifying a reversible cause of dental pain not requiring invasive dental treatment. This will prove helpful considering the many challenges of the public health care system to provide adequate care for oral health problems in these financially disadvantaged, remote, rural regions of central and western Africa. At the same time, inclusion of transient tooth pain into the accepted clinical spectrum of loiasis will help to re-estimate the true burden of disease that loiasis exerts on affected populations in high transmission regions.
